# A guide to open science practices for animal research

**DOI:** 10.1371/journal.pbio.3001810

**Published:** 2022-09-15

**Authors:** Kai Diederich, Kathrin Schmitt, Philipp Schwedhelm, Bettina Bert, Céline Heinl

**Affiliations:** German Federal Institute for Risk Assessment, German Centre for the Protection of Laboratory Animals (Bf3R), Berlin, Germany

## Abstract

Translational biomedical research relies on animal experiments and provides the underlying proof of practice for clinical trials, which places an increased duty of care on translational researchers to derive the maximum possible output from every experiment performed. The implementation of open science practices has the potential to initiate a change in research culture that could improve the transparency and quality of translational research in general, as well as increasing the audience and scientific reach of published research. However, open science has become a buzzword in the scientific community that can often miss mark when it comes to practical implementation. In this Essay, we provide a guide to open science practices that can be applied throughout the research process, from study design, through data collection and analysis, to publication and dissemination, to help scientists improve the transparency and quality of their work. As open science practices continue to evolve, we also provide an online toolbox of resources that we will update continually.

## Abstract 2

Open science has become a buzzword in the scientific community that too often misses the practical application for individual researchers. This Essay, provides a guide to choosing the most appropriate tools to make animal research more transparent.

## Introduction

Over the past decade, the quality of published scientific literature has been repeatedly called into question by the failure of large replication studies or meta-analyses to demonstrate sufficient translation from experimental research into clinical successes [1–5]. At the same time, the open science movement has gained more and more advocates across various research areas. By sharing all of the information collected during the research process with colleagues and with the public, scientists can improve collaborations within their field and increase the reproducibility and trustworthiness of their work [6]. Thus, the International Reproducibility Networks have called for more open research [7].

However, open science practices have not been adopted to the same degree in all research areas. In psychology, which was strongly affected by the so-called reproducibility crisis, the open science movement initiated real practical changes leading to a broad implementation of practices such as preregistration or sharing of data and material [8–10]. By contrast, biomedical research is still lagging behind. Open science might be of high value for research in general, but in translational biomedical research, it is an ethical obligation. It is the responsibility of the scientist to transparently share all data collected to ensure that clinical research can adequately evaluate the risks and benefits of a potential treatment. When Russell and Burch published “The Principles of Humane Experimental Technique” in 1959, scientists started to implement their 3Rs principle to answer the ethical dilemma of animal welfare in the face of scientific progress [11]. By replacing animal experiments wherever possible, reducing the number of animals to a strict minimum, and refining the procedures where animals have still to be used, this ethical dilemma was addressed. However, in recent years, whether the 3Rs principle is sufficient to fully address ethical concerns about animal experiments has been questioned [12].

Most people tolerate the use of animals for scientific purposes only under the basic assumption that the knowledge gained will advance research in crucial areas. This implies that performed experiments are reported in a way that enables peers to benefit from the collected data. However, recent studies suggest that a large proportion of animal experiments are never actually published. For example, scientists working within the European Union (EU) have to write an animal study protocol for approval by the competent authorities of the respective country before performing an animal experiment [13]. In these protocols, scientists have to describe the planned study and justify every animal required for the project. By searching for publications resulting from approved animal study protocols from 2 German University Medical Centers, Wieschowski and colleagues found that only 53% of approved protocols led to a publication after 6 years [14]. Using a similar approach, Van der Naald and colleagues determined a publication rate of 60% at the Utrecht Medical Center [15]. In a follow-up survey, the respective researchers named so-called “negative” or null-hypothesis results as the main cause for not publishing outcomes [15]. The current scientific system is shaped by publishers, funders, and institutions and motivates scientists to publish novel, surprising, and positive results, revealing one of the many structural problems that the numerous efforts towards open science initiatives are targeting. Non-publication not only strongly contradicts ethical values, but also it compromises the quality of published literature by leading to overestimation of effect sizes [16,17]. Furthermore, publications of animal studies too often show poor reporting that strongly impairs the reproducibility, validity, and usefulness of the results [18]. Unfortunately, the idea that negative or equivocal findings can also contribute to the gain of scientific knowledge is frequently neglected.

So far, the scientific community using animals has shown limited resonance to the open science movement. Due to the strong controversy surrounding animal experiments, scientists have been reluctant to share information on the topic. Additionally, translational research is highly competitive and researchers tend to be secretive about their ideas until they are ready for publication or patent [19,20]. However, this missing openness could also point to a lack of knowledge and training on the many open science options that are available and suitable for animal research. Researchers have to be convinced of the benefits of open science practices, not only for science in general, but also for the individual researcher and each single animal. Yet, the key players in the research system are already starting to value open science practices. An increasing number of journals request open sharing of data, funders pay for open access publications and institutions consider open science practices in hiring decisions. Open science practices can improve the quality of work by enabling valuable scientific input from peers at the early stages of research projects. Furthermore, the extended communication that open science practices offer can draw attention to research and help to expand networks of collaborators and lead to new project opportunities or follow-up positions. Thus, open science practices can be a driver for careers in academia, particularly those of early career researchers.

Beyond these personal benefits, improving transparency in translational biomedical research can boost scientific progress in general. By bringing to light all the recorded research outputs that until now have remained hidden, the publication bias and the overestimation of effect sizes can be reduced [17]. Large-scale sharing of data can help to synthesize research outputs in preclinical research that will enable better decision-making for clinical research. Disclosing the whole research process will help to uncover systematic problems and support scientists in thoroughly planning their studies. In the long run, we predict that the implementation of open science practices will lead to the use of fewer animals in unintentionally repeated experiments that previously showed unreported negative results or in the establishment of methods by avoiding experimental dead ends that are often not published. More collaborations and sharing of materials and methods can further reduce the number of animal experiments used for the implementation of new techniques.

**Fig 1:**
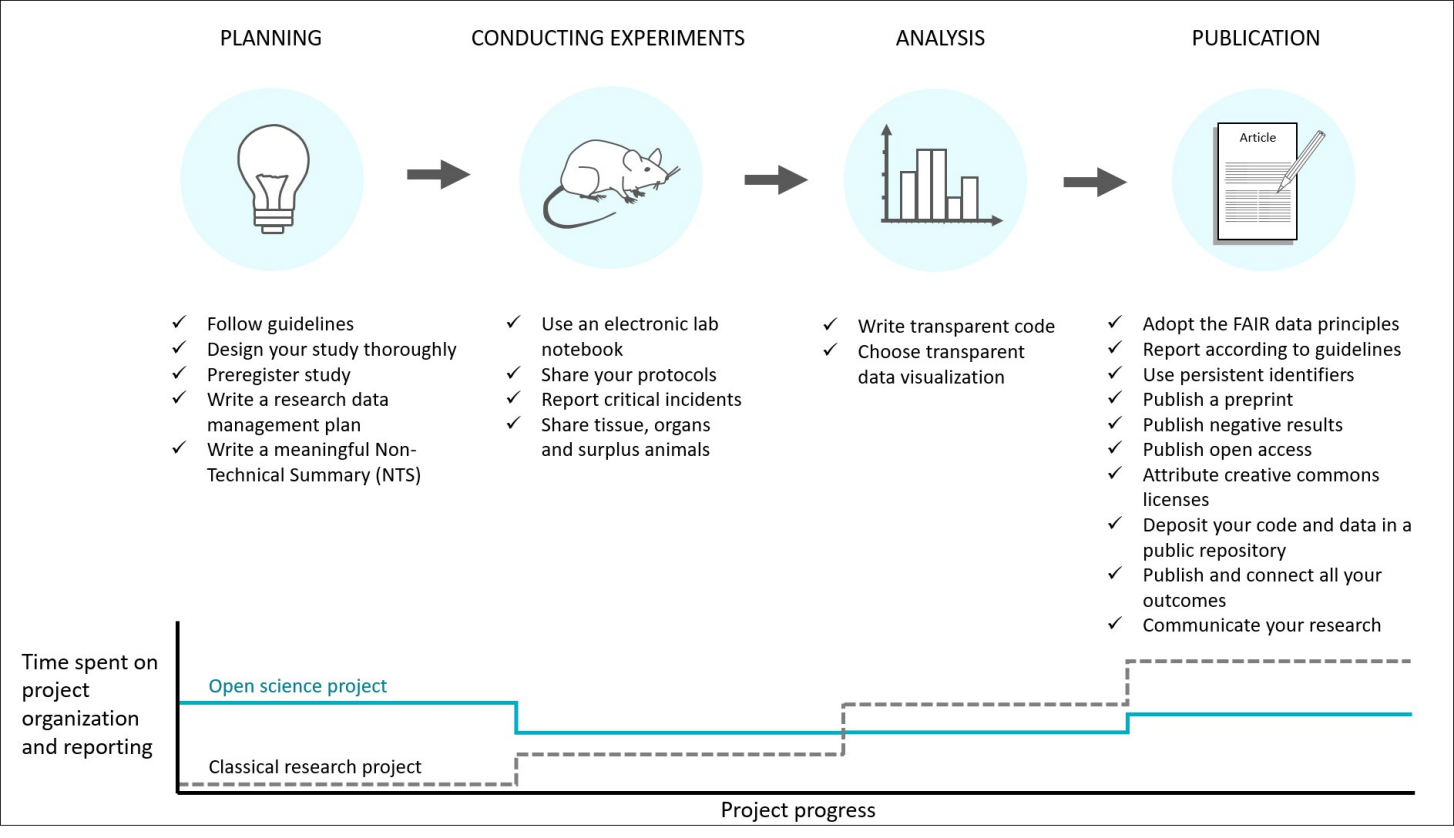
Using open science practices throughout translational research studies. Application of open science practices at each step of the research process can maximize the impact of performed animal experiments. The implementation of these practices will lead to less time pressure at the end of a project. Due to the connection of most of these open science practices, spending more time in the planning phase and during the conduction of experiments will save time during the data analysis and publication of the study. Indeed, consulting reporting guidelines early on, preregistering a statistical plan, and writing down crucial experimental details in an electronic lab notebook, will strongly accelerate the writing of a manuscript. If protocols or even electronic lab notebooks were made public, just citing these would simplify the writing of publications. Similarly, if a data management plan is well designed before starting data collection, analyzing, and depositing data in a public repository, as is increasingly required, will be fast. NTS, non-technical summary.

Open science can and should be implemented at each step of the research process (Fig 1). A vast number of tools are already provided that were either directly conceptualized for animal research or can be adapted easily. In this Essay, we provide an overview of open science tools that improve transparency, reliability, and animal welfare in translational in vivo biomedical research by supporting scientists to clearly communicate their research and by supporting collaborative working. Table 1 lists the most prominent open science tools we discuss, together with their respective links. We have structured this Essay to guide you through which tools can be used at each stage of the research process, from planning and conducting experiments, through to analyzing data and communicating the results. However, many of these tools can be used at many different steps. Table 1 has been deposited on Zenodo and will be updated continuously [21].

**Table 1:** Open science toolbox for translational biomedical research.

	Open science practice	Specific tools	Corresponding links
PLANNING	Guidelines	PREPARE Guidelines	https://norecopa.no/prepare
		UKRN Primers	https://www.ukrn.org/primers/
		ARRIVE Guidelines	https://arriveguidelines.org/
	Design your study thoroughly	Literature and tools for the integration of sex and gender in research	https://cihr-irsc.gc.ca/e/50836.html
		Sample size calculator G*Power	https://www.psychologie.hhu.de/arbeitsgruppen/allgemeine-psychologie-und-arbeitspsychologie/gpower
		Software package for R for the statistical planning for animal research	https://invivostat.co.uk
		Tool for the randomization for experimental planning	https://www.randomizer.org/
		Creating a sharable scheme with the EDA	https://eda.nc3rs.org.uk/
	Preregistration	Preclinicaltrials.eu	https://www.preclinicaltrials.eu/
		Animalstudyregistry.org	https://www.animalstudyregistry.org
		OSF Registry	https://osf.io/registries
		List of journals offering registered reports	https://www.cos.io/initiatives/registered-reports
	Writing a research data management plan	Research data management checklist from the Harvard Medical School	https://datamanagement.hms.harvard.edu/plan-design
		Research data management toolkit from JISC	https://www.jisc.ac.uk/full-guide/rdm-toolkit
		DMPTool	https://dmptool.org/
		DMPonline	https://dmponline.dcc.ac.uk/
	Writing a non-technical summary	Alures: the Europe-wide NTS database	https://webgate.ec.europa.eu/envdataportal/web/resources/alures/submission/nts/list
		Guide to writing non-technical summaries from UAR	https://concordatopenness.org.uk/guide-to-writing-non-technical-summaries
CONDUCTING EXPERIMENTS	Using an electronic lab notebook	Table of ELNs with features	https://zenodo.org/record/4723753
	Sharing protocols	Protocols.io	https://www.protocols.io/
		Protocol exchange	https://protocolexchange.researchsquare.com/
		Bio-protocol	https://bio-protocol.org/Default.aspx
	Reporting critical incidents	Critical incident reporting CIRS-LAS	https://www.cirs-las.de/home
	Sharing animals, organs and tissue	Online sharing platform for organs and tissues	www.animatch.eu
		Open-source software to facilitate intuitional organ sharing	https://github.com/hdinkel/anishare
		Searchable online data base of mouse strain resources from multiple repositories	http://www.findmice.org/index
ANALYSIS	Writing transparent code	Jupyter Notebooks	https://jupyter.org/
		GitHub	https://github.com/
		R	https://www.r-project.org/
	Choosing transparent data visualization	Paper with a list of free tools for more transparent data visualization	https://www.ahajournals.org/doi/epub/10.1161/CIRCULATIONAHA.118.037777
		Tool to check graph accessibility for color blind persons	https://colororacle.org/index.html
PUBLICATION	Adopting the FAIR data principles	A guide on how to implement the FAIR data principles	https://www.go-fair.org/how-to-go-fair/
	Using field specific reporting guidelines	ARRIVE Guidelines	https://arriveguidelines.org/
		Tool from the EQUATOR Network to find specific reporting guidelines	https://www.equator-network.org/reporting-guidelines/
		MERIDIAN–collection of all reporting guidelines involving animals	https://meridian.cvm.iastate.edu/
	Using persistent identifiers	Unmistakably identify publications: DOI	https://www.doi.org/
		Unmistakably identify authors: ORCID-ID	https://orcid.org/
		Unmistakably identify resources: RRID	https://scicrunch.org/resources
		Unmistakably identify mouse lines: The MGI	http://www.informatics.jax.org/mgihome/nomen/index.shtml
	Publishing preprints	Searchable database of preprint servers	https://asapbio.org/preprint-servers
		BioRxiv	https://www.biorxiv.org/
		MedRxiv	https://www.medrxiv.org/
		OSF preprints	https://osf.io/preprints/
	Publishing negative results	fiddle—file drawer data liberation effort to identify a way of publication for null results	https://s-quest.bihealth.org/fiddle/
	Publishing open access	Journals listed by the DOAJ	https://doaj.org/
		Gold Open Access: List of open access biomedical journals	https://s-quest.bihealth.org/OAPositiveList/
		Green Open Access: List of open Access repositories OpenDOAR:	https://v2.sherpa.ac.uk/cgi/search/repository/advanced
		Find open access policies of journals and publishers: Sherpa Romeo	https://v2.sherpa.ac.uk/romeo/search.html
	Depositing code and data in public repositories	Finding a research field specific repository	https://www.re3data.org/browse/by-subject/ https://beta.fairsharing.org/
		Open Science Framework	https://osf.io/
		Figshare	https://figshare.com/
		Dryad	https://datadryad.org/stash
		Zenodo	https://zenodo.org/
	Attributing creative commons licenses	Attributing the adequate creative commons license	https://creativecommons.org/choose/
	Publishing and connecting all outcomes	Open Science Framework	https://osf.io/
	Communicating research	Twitter	https://twitter.com/
		ResearchGate	https://www.researchgate.net/

A copy of this table has been deposited at Zenodo and will be updated continuously 10.5281/zenodo.6497559.

DOAJ, Directory of Open Access Journals; DOI, digital object identifier; EDA, Experimental Design Assistant; MGI, Mouse Genome Informatics; RRID, Research Resource Identifier.

### Planning the study

Transparent practices can be adopted at every stage of the research process. However, to ensure full effectivity, it is highly recommended to engage in detailed planning before the start of the experiment. This can prevent valuable time from being lost at the end of the study due to careless decisions being made at the beginning. Clarifying data management at the start of a project can help avoiding filing chaos that can be very time consuming to untangle. Keeping clear track of a project and study design will also help if new colleagues are included later on in the project or if entire project parts are handed over. In addition, all texts written on the rationale and hypothesis of the study or method descriptions, or design schemes created during the planning phase can be used in the final publications (Fig 1). Similarly, information required for preregistration of animal studies or for reporting according to the ARRIVE guidelines are an extension of the details required for ethical approval [22,23]. Thus, the time burden within the planning phase is often overestimated. Furthermore, the thorough planning of experiments can avoid the unnecessary use of animals by preventing wrong avenues from being pursued.

Implementing open scientific practices at the beginning of a project does not mean that the idea and study plan must be shared immediately, but rather is critical for making the entire workflow transparent at the end of the project. However, optional early sharing of information can enable peers to give feedback on the study plan. Studies potentially benefit more from this a priori input than they would from the classical a posteriori peer-review process.

### Guidelines

Most people perceive guidelines as advice that instructs on how to do something. However, it is sometimes useful to consider the term in its original meaning; “the line that guides us”. In this sense, following guidelines is not simply fulfilling a duty, but is a process that can help to design a sound research study and, as such, guidelines should be consulted at the planning stage of a project. The PREPARE guidelines are a list of important points that should be thought-out before starting a study involving animal experiments in order to reduce the waste of animals, promote alternatives, and increase the reproducibility of research and testing [24]. The PREPARE checklist helps to thoroughly plan a study and focuses on improving the communication and collaboration between all involved participants of the study (i.e., animal caretakers and scientists). Indeed, open science begins with the communication within a research facility. It is currently available in 33 languages and the responsible team from Norecopa, Norway’s 3R-center, takes requests for translations into further languages.

The UK Reproducibility Network has also published several guiding documents (primers) on important topics for open and reproducible science. These address issues such as data sharing [25], open access [26], open code and software [27], and preprints [28], as well as preregistration and registered reports [27]. Consultation of these primers is not only helpful in the relevant phases of the experiment but is also encouraged in the planning phase.

Although the ARRIVE guidelines are primarily a reporting guideline specifically designed for preparing a publication containing animal data, they can also support researchers when planning their experiments [22,23]. Going through the ARRIVE website, researchers will find tools and explanations that can support them in planning their experiments [29]. Consulting the ARRIVE checklist at the beginning of a project can help in deciding what details need to be documented during conduction of the experiments. This is particularly advisable, given that compliance to ARRIVE is still poor [18].

### Experimental design

To maximize the validity of performed experiments and the knowledge gained, designing the study well is crucial. It is important that the chosen animal species reflects the investigated disease well and that basic characteristics of the animal, such as sex or age, are considered carefully [30]. The Canadian Institutes of Health Research provides a collection of resources on the integration of sex and gender in biomedical research with animals, including tips and tools for researchers and reviewers [31]. Additionally, it is advisable to avoid unnecessary standardization of biological and environmental factors that can reduce the external validity of results [32]. Meticulous statistical planning can further optimize the use of animals. Free to use online tools for calculating sample sizes such as G*Power or the inVivo software package for R can further support animal researchers in designing their statistical plan [33,34]. Randomization for the allocation of groups can be supported with specific tools for scientists like Research Randomizer, but also by simple online random number generators [35]. Furthermore, it might be advisable when designing the study to incorporate pathological analyses into the experimental plan. Optimal planning of tissue collection, performance of pathological procedures according to accepted best practices, and use of optimal pathological analysis and reporting methods can add some extra knowledge that would otherwise be lost. This can improve the reproducibility and quality of translational biomedicine, especially, but not exclusively, in animal studies with morphological endpoints. In all animal studies, unexpected deaths in experimental animals can occur and be the cause of lost data or missed opportunities to identify health problems [36,37].

To support researchers in designing their animal research, the National Centre for the Replacement, Refinement and Reduction of Animals in Research (NC3Rs) has also developed the Experimental Design Assistant (EDA) [38,39]. This online tool helps researchers to better structure in vivo research by creating detailed schemes of the study design. It provides feedback on the entered design, drawing researcher’s attention to crucial decisions in the project. The resulting schemes can be used to transparently share the study design by uploading it into a study preregistration, enclosing it in a grant application, or submitting it with a final manuscript. The EDA can be used for different study designs in diverse scenarios and helps to communicate researcher plans to others [40]. The EDA might be particularly of interest to clarify very complex study designs involving multiple experimental groups. Working with the EDA might appear rather complex in the beginning, but the NC3R provides regular webinars that can help to answer any questions that arise.

### Preregistration

Preregistration is an effective tool to improve the quality and transparency of research. To preregister their work, scientists must determine crucial details of the study before starting any experiment. Changes occurring during a study can be outlined at the end. A preregistered study plan should include at least the hypothesis and determine all the parameters that are known in advance. A description of the planned study design and statistical analysis will enable reviewers and peers to better retrace the workflow. It can prevent the intentional use of the flexibility of analysis to reach *p*-values under a certain significance level (e.g., p-hacking or HARKing (Hypothesizing After Results are Known)). With preregistration, scientists can also claim their idea at an early stage of their research with a citable individual identifier that labels the idea as their own. Some open preregistration platforms also provide a digital object identifier (DOI), which makes the registered study citable. Three public registries actively encourage the preregistration of animal studies conducted around the world: OSF registry, preclinicaltrials.eu, and animalstudyregistry.org [41–45]. Scientists can choose the registry according to their needs. Preregistering a study in a public registry supports scientists in planning their study and later to critically reevaluate their own work and assess its limitations and potentials.

As an alternative to public registries, researchers can also submit their study plan to one of hundreds of journals already publishing registered reports, including many journals open to animal research [8]. A submitted registered report passes 2 steps of peer review. In the first step, reviewers comment on the idea and the study design. After an “in-principle-acceptance,” researchers can conduct their study as planned. If the authors conduct the experiments as described in the accepted study protocol, the journal will publish the final study regardless of the outcome. This might be an attractive option, especially for early career researchers, as a manuscript is published at the beginning of a project with the guarantee of a future final publication.

The benefits of preregistration can already be observed in clinical research, where registration has been mandatory for most trials for more than 20 years. Preregistration in clinical research has helped to make known what has been tested and not just what worked and was published, and the implementation of trial registration has strongly reduced the number of publications reporting significant treatment effects [46]. In animal research, with its unrealistically high percentage of positive results, preregistration seems to be particularly worthwhile.

### Research data management

To get the most out of performed animal experiments, effective sharing of data at the end of the study is essential. Sharing research data optimally is complex and needs to be prepared in advance. Thus, data management can be seen as one part of planning a study thoroughly. Many funders have recognized the value of the original research data and request a data management plan from applicants in advance [25,47]. Various freely available tools such as DMPTool or DMPonline already exist to design a research data management plan that complies to the requirements of different funders [48,49]. The data management plan defines the types of data collected and describes the handling and names responsible persons throughout the data lifecycle. This includes collecting the data, analyzing, archiving, and sharing it. Finally, a data management plan enables long-term access and the possibility for reuse by peers. Developing such a plan, whether it is required by funders or not, will later simplify the application of the FAIR data principle (see section on the FAIR data principle). The Longwood Medical Area Research Data Management Working Group from the Harvard Medical School developed a checklist to assist researchers in optimally managing their data throughout the data lifecycle [50]. Similarly, the Joint Information Systems Committee (JISC) provides a great research data management toolkit including a checklist for researchers planning their project [51]. Consulting this checklist in the planning phase of a project can prevent common errors in research data management.

### Non-technical project summary

One instrument specifically conceived to create transparency on animal research for the general public is the so-called non-technical project summary (NTS). All animal protocols approved within the EU must be accompanied by these comprehensible summaries. NTSs are intended to inform the public about ongoing animal experiments. They are anonymous and include information on the objectives and potential benefits of the project, the expected harm, the number of animals, the species, and a statement of compliance with the requirements of the 3Rs principle. However, beyond simply informing the public, NTSs can also be used for meta-research to help identify new research areas with an increased need for new 3R technologies [52,53]. NTSs become an excellent tool to appropriately communicate the scientific value of the approved protocol and for meta-scientists to generate added value by systematically analyzing theses summaries if they fulfill a minimum quality threshold [54,55]. In 2021, the EU launched the ALURES platform (Table 1), where NTSs from all member states are published together, opening the opportunities for EU-wide meta-research. NTSs are, in contrast to other open science practices, mandatory in the EU. However, instead of thinking of them as an annoying duty, it might be worth thoroughly drafting the NTS to support the goals of more transparency towards the public, enabling an open dialogue and reducing extreme opinions.

### Conducting the experiments

Once the experiments begin, documentation of all necessary details is essential to ensure the transparency of the workflow. This includes methodological details that are crucial for replicating experiments, but also failed attempts that could help peers to avoid experiments that do not work in the future. All information should be stored in such a way that it can be found easily and shared later. In this area, many new tools have emerged in recent years (Table 1). These tools will not only make research transparent for colleagues, but also help to keep track of one’s own research and improve internal collaboration.

### Electronic laboratory notebooks

Electronic laboratory notebooks (ELNs) are an important pillar of research data management and open science. ELNs facilitate the structured and harmonized documentation of the data generation workflow, ensure data integrity, and keep track of all modifications made to the original data based on an audit trail option. Moreover, ELNs simplify the sharing of data and support collaborations within and outside the research group. Methodological details and research data become searchable and traceable. There is an extensive amount of literature providing advice on the selection and the implementation process of an ELN depending on the specific needs and research area and its discussion would be beyond the scope of this Essay [56–58]. Some ELNs are connected to a laboratory information management system (LIMS) that provides an animal module supporting the tracking of animal details [59]. But as research involving animals is highly heterogeneous, this might not be the only decision point and we cannot recommend a specific ELN that is suitable for all animal research.

ELNs are already established in the pharmaceutical industry and their use is on the rise among academics as well. However, due to concerns around costs for licenses, data security, and loss of flexibility, many research institutions still fear the expenses that the introduction of such a system would incur [56]. Nevertheless, an increasing number of academic institutions are implementing ELNs and appreciating the associated benefits [60]. If your institution already has an ELN, it might be easiest to just use the option available in the research environment. If not, the Harvard Medical School provides an extensive and updated overview of various features of different ELNs that can support scientists in choosing the appropriate one for their research [61]. There are many commercial ELN products, which may be preferred when the administrative workload should be outsourced to a large extent. However, open-source products such as eLabFTW or open BIS provide a greater opportunity for customization to meet specific needs of individual research institutions [62–64]. A huge number of options are available depending on the resources and the features required. Some scientists might prefer generic note taking tools such as Evernote or just a simple Word document that offers infinite flexibility, but specific ELNs can further support good record keeping practice by providing immutability, automated backups, standardized methods, and protocols to follow. Clearly defining the specific requirements expected might help to choose an adequate system that would improve the quality of the record compared to classical paper laboratory notebooks.

### Sharing protocols

Adequate sharing of methods in translational biomedical sciences is key to reproducibility. Several repositories exist that simplify the publication and exchange of protocols. Writing down methods at the end of the project bears the risk that crucial details might be missing [65]. On protocols.io, scientists can note all methodological details of a procedure, complete them with uploaded documents, and keep them for personal use or share them with collaborators [66]. Authors can also decide at any point in time to make their protocol public. Protocols published on protocols.io receive a DOI and become citable; they can be commented on by peers and adapted according to the needs of the individual researcher. Protocol.io files from established protocols can also be submitted together with some context and sample datasets to *PLOS ONE*, where it can be peer-reviewed and potentially published [67,68]. Depending on the affiliation of the researchers to academia or industry and on an internal or public sharing of files, protocols.io can be free of charge or come with costs. Other journals also encourage their authors to deposit their protocols in a freely accessible repository, such as protocol exchange from Nature portfolio [69]. Another option might be to separately submit a protocol that was validated by its use in an already published research article to an online and peer-reviewed journal specific for research protocols, such as Bio-Protocol. A multitude of journals, including eLife and Science already collaborate with Bio-Protocol and recommend authors to publish the method in Bio-Protocol [70]. Bio-Protocol has no submission fees and is freely available to all readers. Both protocols.io and Bio-Protocol allow the illustration of complex scientific methods by uploading videos to published protocols. In addition, protocols can be deposited in a general research repository such as the Open Science Framework (OSF repository) and referenced in appropriate publications.

### Sharing critical incidents

Sharing critical or even adverse events that occur in the context of animal experimentation can help other scientists to avoid committing the same mistakes. The system of sharing critical incidents is already established in clinical practice and helps to improve medical care [71,72]. The online platform critical incident reporting system in laboratory animal science (CIRS-LAS) represents the first preclinical equivalent to these clinical systems [73]. With this web-based tool, critical incidents in animal research can be reported anonymously without registration. An expert panel helps to analyze the incident to encourage an open dialogue. Critical incident reporting is still very marginal in animal research and performed procedures are very variable. These factors make a systemic analysis and a targeted search of incidence difficult. However, it may be of special interest for methods that are broadly used in animal research such as anesthesia. Indeed, a broad feed of this system with data on errors occurring in standard procedures today could help avoid critical incidences in the future and refine animal experiments.

### Sharing animals, organs, and tissue

When we think about open science, sharing results and data are often in focus. However, sharing material is also part of a collaborative and open research culture that could help to greatly reduce the number of experimental animals used. When an animal is killed to obtain specific tissue or organs, the remainder is mostly discarded. This may constitute a wasteful practice, as surplus tissue can be used by other researchers for different analyses. More animals are currently killed as surplus than are used in experiments, demonstrating the potential for sharing these animals [74,75].

Sharing information on generated surplus is therefore not only economical, but also an effective way to reduce the number of animals used for scientific purposes. The open-source software Anishare is a straightforward way for breeders of genetically modified lines to promote their surplus offspring or organs within an institution [76]. The database AniMatch (Table 1) connects scientists within Europe who are offering tissue or organs with scientists seeking this material. Scientists already sharing animal organs can support this process by describing it in publications and making peers aware of this possibility [77]. Specialized research communities also allow sharing of animal tissue or animal-derived products worldwide that are typically used in these fields on a collaborative basis via the SEARCH-framework [78,79]. Depositing transgenic mice lines into one of several repositories for mouse strains can help to further minimize efforts in producing new transgenic lines and most importantly reduce the number of surplus animals by supporting the cryoconservation of mouse lines. The International Mouse Strain Resource (IMSR) can be used to help find an adequate repository or to help scientists seeking a specific transgenic line find a match [80].

### Analyzing the data

Animal researchers have to handle increasingly complex data. Imaging, electrophysiological recording, or automated behavioral tracking, for example, produce huge datasets. Data can be shared as raw numerical output but also as images, videos, sounds, or other forms from which raw numerical data can be generated. As the heterogeneity and the complexity of research data increases, infinite possibilities for analysis emerge. Transparently reporting how the data were processed will enable peers to better interpret reported results. To get the most out of performed animal experiments, it is crucial to allow other scientists to replicate the analysis and adapt it to their research questions. It is therefore highly recommended to use formats and tools during the analysis that allow a straightforward exchange of code and data later on.

### Transparent coding

The use of non-transparent analysis codes have led to a lack of reproducibility of results [81]. Sharing code is essential for complex analysis and enables other researchers to reproduce results and perform follow-up studies, and citable code gives credit for the development of new algorithms (Table 1). Jupyter Notebooks are a convenient way to share data science pipelines that may use a variety of coding languages, including like Python, R or Matlab, and also share the results of analyses in the form of tables, diagrams, images, and videos. Notebooks contain source code and can be published or collaboratively shared on platforms like GitHub or GitLab, where version control of source code is implemented. The data-archiving tool Zenodo can be used to archive a repository on GitHub and create a DOI for the archive. Thereby contents become citable. Using free and open-source programming language like R or Python will increase the number of potential researchers that can work with the published code. Best practice for research software is to publish the source code with a license that allows modification and redistribution.

### Choice of data visualization

Choosing the right format for the visualization of data can increase its accessibility to a broad scientific audience and enable peers to better judge the validity of the results. Studies based on animal research often work with very small sample sizes. Visualizing these data in histograms may lead to an overestimation of the outcomes. Choosing the right dot plots that makes all recorded points visible and at the same time focusses on the summary instead of the individual points can further improve the intuitive understanding of a result. If the sample size is too low, it might not be meaningful to visualize error bars. A variety of freely available tools already exists that can support scientists in creating the most appropriate graphs for their data [82]. In particular, when representing microscopy results or heat maps, it should be kept in mind that a large part of the population cannot perceive the classical red and green representation [83]. Opting for the color-blind safe color maps and checking images with free tools such as color oracle (Table 1) can increase the accessibility of graphs. Multiple journals have already addressed flaws in data visualization and have introduced new policies that will accelerate the uptake of transparent representation of results.

### Publication of all study outcomes

Open science practices have received much attention in the past few years when it comes to publication of the results. However, it is important to emphasize that although open science tools have their greatest impact at the end of the project, good study preparation and sharing of the study plan and data early on can greatly increase the transparency at the end.

### The FAIR data principle

To maximize the impact and outcome of a study, and to make the best long-term use of data generated through animal experiments, researchers should publish all data collected during their research according to the FAIR data principle. That means the data should be findable, accessible, interoperable, and reusable. The FAIR principle is thus an extension of open access publishing. Data should not only be published without paywalls or other access restrictions, but also in such a manner that they can be reused and further processed by others. For this, legal as well as technical requirements must be met by the data. To achieve this, the GoFAIR initiative has developed a set of principles that should be taken into account as early as at the data collection stage [49,84]. In addition to extensively described and machine-readable metadata, these principles include, for example, the application of globally persistent identifiers, the use of open file formats, and standardized communication protocols to ensure that humans and machines can easily download the data. A well-chosen repository to upload the data is then just the final step to publish FAIR data.

FAIR data can strongly increase the knowledge gained from performed animal experiments. Thus, the same data can be analyzed by different researchers and could be combined to obtain larger sample sizes, as already occurs in the neuroimaging community, which works with comparable datasets [85]. Furthermore, the sharing of data enables other researchers to analyze published datasets and estimate measurement reliabilities to optimize their own data collection [86,87]. This will help to improve the translation from animal research into clinics and simultaneously reduce the number of animal experiment in future.

### Reporting guidelines

In preclinical research, the ARRIVE guidelines are the current state of the art when it comes to reporting data based on animal experiments [22,23]. The ARRIVE guidelines have been endorsed by more than 1,000 journals who ask that scientists comply with them when reporting their outcomes. Since the ARRIVE guidelines have not had the expected impact on the transparency of reporting in animal research publications, a more rigorous update has been developed to facilitate their application in practice (ARRIVE 2.0 [23]). We believe that the ARRIVE guidelines can be more effective if they are implemented at a very early stage of the project (see section on guidelines). Some more specialized reporting guidelines have also emerged for individual research fields that rely on animal studies, such as endodontology [88]. The equator network collects all guidelines and makes them easily findable with their search tool on their website (Table 1). MERIDIAN also offers a 1-stop shop for all reporting guidelines involving the use of animals across all research sectors [89]. It is thus worth checking for new reporting guidelines before preparing a manuscript to maximize the transparency of described experiments.

### Identifiers

Persistent identifiers for published work, authors, or resources are key for making public data findable by search engines and are thus a prerequisite for compliance to FAIR data principles. The most common identifier for publications will be a DOI, which makes the work citable. A DOI is a globally unique string assigned by the International DOI Foundation to identify content permanently and provide a persistent link to its location on the Internet. An ORCID ID is used as a personal persistent identifier and is recommendable to unmistakably identify an author (Table 1). This will avoid confusions between authors with the same name or in the case of name changes or changes of affiliation. Research Resource Identifiers (RRID) are unique ID numbers that help to transparently report research resources. RRID also apply to animals to clearly identify the species used. RRID help avoid confusion between different names or changing names of genetic lines and, importantly, make them machine findable. The RRID Portal helps scientists find a specific RRID or create one if necessary (Table 1). In the context of genetically altered animal lines, correct naming is key. The Mouse Genome Informatics (MGI) Database is the authoritative source of official names for mouse genes, alleles, and strains ([90]).

### Preprint publication

Preprints have undergone unprecedented success, particularly during the height of the Coronavirus Disease 2019 (COVID-19) pandemic when the need for rapid dissemination of scientific knowledge was critical. The publication process for scientific manuscripts in peer-reviewed journals usually requires a considerable amount of time, ranging from a few months to several years, mainly due to the lengthy review process and inefficient editorial procedures [91,92]. Preprints typically precede formal publication in scientific journals and, thus, do not go through a peer review process, thus, facilitating the prompt open dissemination of important scientific findings within the scientific community. However, submitted papers are usually screened and checked for plagiarism. Preprints are assigned a DOI so they can be cited. Once a preprint is published in a journal, its status is automatically updated on the preprint server. The preprint is linked to the publication via CrossRef and mentioned accordingly on the website of the respective preprint platform.

After initial skepticism, most publishers now allow papers to be posted on preprint servers prior to submission. An increasing number of journals even allow direct submission of a preprint to their peer review process. The US National Institutes of Health and the Wellcome Trust, among other funders, also encourage prepublication and permit researchers to cite preprints in their grant applications. There are now numerous preprint repositories for different scientific disciplines. BioASAP provides a searchable database for preprint servers that can help in identifying the one that best matches an individual’s needs [93]. The most popular repository for animal research is bioRxiv, which is hosted by the Cold Spring Harbor Laboratory (Table 1).

The early exchange of scientific results is particularly important for animal research. This acceleration of the publication process can help other scientists to adapt their research or could even prevent animal experiments if other scientists become aware that an experiment has already been done before starting their own. In addition, preprints can help to increase the visibility of research. Journal articles that have a corresponding preprint publication have higher citation and Altmetric counts than articles without preprint [94]. In addition, the publication of preprints can help to combat publication bias, which represents a major problem in animal research [16]. Since journals and readers prioritize cutting-edge studies with positive results over inconclusive or negative results, researchers are reluctant to invest time and money in a manuscript that is unlikely to be accepted in a high-impact journal.

In addition to the option of publishing as preprint, other alternative publication formats have recently been introduced to facilitate the publication of research results that are hard to publish in traditional peer-reviewed journals. These include micro publications, data repositories, data journals, publication platforms, and journals that focus on negative or inconclusive results. The tool fiddle can support scientists in choosing the right publication format [95,96].

### Open access publication

Publishing open access is one of the most established open science strategies. In contrast to the FAIR data principle, the term open access publication refers usually to the publication of a manuscript on a platform that is accessible free of charge—in translational biomedical research, this is mostly in the form of a scientific journal article. Originally, publications accessible free of charge were the answer to the paywalls established by renowned publishing houses, which led to social inequalities within and outside the research system. In translational biomedical research, the ethical aspect of urgently needed transparency is another argument in favor of open access publication, as these studies will not only be findable, but also internationally readable.

There are different ways of open access publishing; the 2 main routes are gold open access and green open access. Numerous journals offer now gold open access. It refers to the immediate and fully accessible publication of an article. The Directory of Open Access Journals (DOAJ) provides a complete and updated list for high-quality, open access, and peer-reviewed journals [97]. Charité–Universitätsmedizin Berlin offers a specific tool for biomedical open access journals that supports animal researchers to choose an appropriate journal [49]. In addition, the Sherpa Romeo platform is a straightforward way to identify publisher open access policies on a journal-by-journal basis, including information on preprints, but also on licensing of articles [51]. Hybrid open access refers to openly accessible articles in otherwise paywalled journals. By contrast, green open access refers to the publication of a manuscript or article in a repository that is mostly operated by institutions and/or universities. The publication can be exclusively on the repository or in combination with a publisher. In the quality-assured, global Directory of Open Access Repositories (openDOAR), scientists can find thousands of indexed open access repositories [49]. The publisher often sets an embargo during which the authors cannot make the publication available in the repository, which can restrict the combined model. It is worth mentioning that gold open access is usually more expensive for the authors, as they have to pay an article processing charge. However, the article’s outreach is usually much higher than the outreach of an article in a repository or available exclusively as subscription content [98]. Diamond open access refers to publications and publication platforms that can be read free of charge by anyone interested and for which no costs are incurred by the authors either. It is the simplest and fairest form of open access for all parties involved, as no one is prevented from participating in scientific discourse by payment barriers. For now, it is not as widespread as the other forms because publishers have to find alternative sources of revenue to cover their costs.

As social media and the researcher’s individual public outreach are becoming increasingly important, it should be remembered that the accessibility of a publication should not be confused with the licensing under which the publication is made available. In order to be able to share and reuse one’s own work in the future, we recommend looking for journals that allow publications under the Creative Commons licenses CC BY or CC BY-NC. This also allows the immediate combination of gold and green open access.

### Creative commons licenses

Attributing Creative Commons (CC) licenses to scientific content can make research broadly available and clearly specifies the terms and conditions under which people can reuse and redistribute the intellectual property, namely publications and data, while giving the credit to whom it deserves [49]. As the laws on copyright vary from country to country and law texts are difficult to understand for outsiders, the CC licenses are designed to be easily understandable and are available in 41 languages. This way, users can easily avoid accidental misuse. The CC initiative developed a tool that enables researchers to find the license that best fits their interests [49]. Since the licenses are based on a modular concept ranging from relatively unrestricted licenses (CC BY, free to use, credit must be given) to more restricted licenses (CC BY-NC-ND, only free to share for non-commercial purposes, credit must be given), one can find an appropriate license even for the most sensitive content. Publishing under an open CC license will not only make the publication easy to access but can also help to increase its reach. It can stimulate other researchers and the interested public to share this article within their network and to make the best future use of it. Bear in mind that datasets published independently from an article may receive a different CC license. In terms of intellectual property, data are not protected in the same way as articles, which is why the CC initiative in the United Kingdom recommends publishing them under a CC0 (“no rights reserved”) license or the Public Domain Mark. This gives everybody the right to use the data freely. In an animal ethics sense, this is especially important in order to get the most out of data derived from animal experiments.

### Data and code repositories

Sharing research data is essential to ensure reproducibility and to facilitate scientific progress. This is particularly true in animal research and the scientific community increasingly recognizes the value of sharing research data. However, even though there is increasing support for the sharing of data, researchers still perceive barriers when it comes to doing so in practice [99–101]. Many universities and research institutions have established research data repositories that provide continuous access to datasets in a trusted environment. Many of these data repositories are tied to specific research areas, geographic regions, or scientific institutions. Due to the growing number and overall heterogeneity of these repositories, it can be difficult for researchers, funding agencies, publishers, and academic institutions to identify appropriate repositories for storing and searching research data.

Recently, several web-based tools have been developed to help in the selection of a suitable repository. One example is Re3data, a global registry of research data repositories that includes repositories from various scientific disciplines. The extensive database can be searched by country, content (e.g., raw data, source code), and scientific discipline [49]. A similar tool to help find a data archive specific to the field is FAIRsharing, based at Oxford University [102]. If there is no appropriate subject-specific data repository or one seems unsuitable for the data, there are general data repositories, such as Open Science Framework, figshare, Dryad, or Zenodo. To ensure that data stored in a repository can be found, a DOI is assigned to the data. Choosing the right license for the deposited code and data ensures that authors get credit for their work.

### Publication and connection of all outcomes

**Fig 2:**
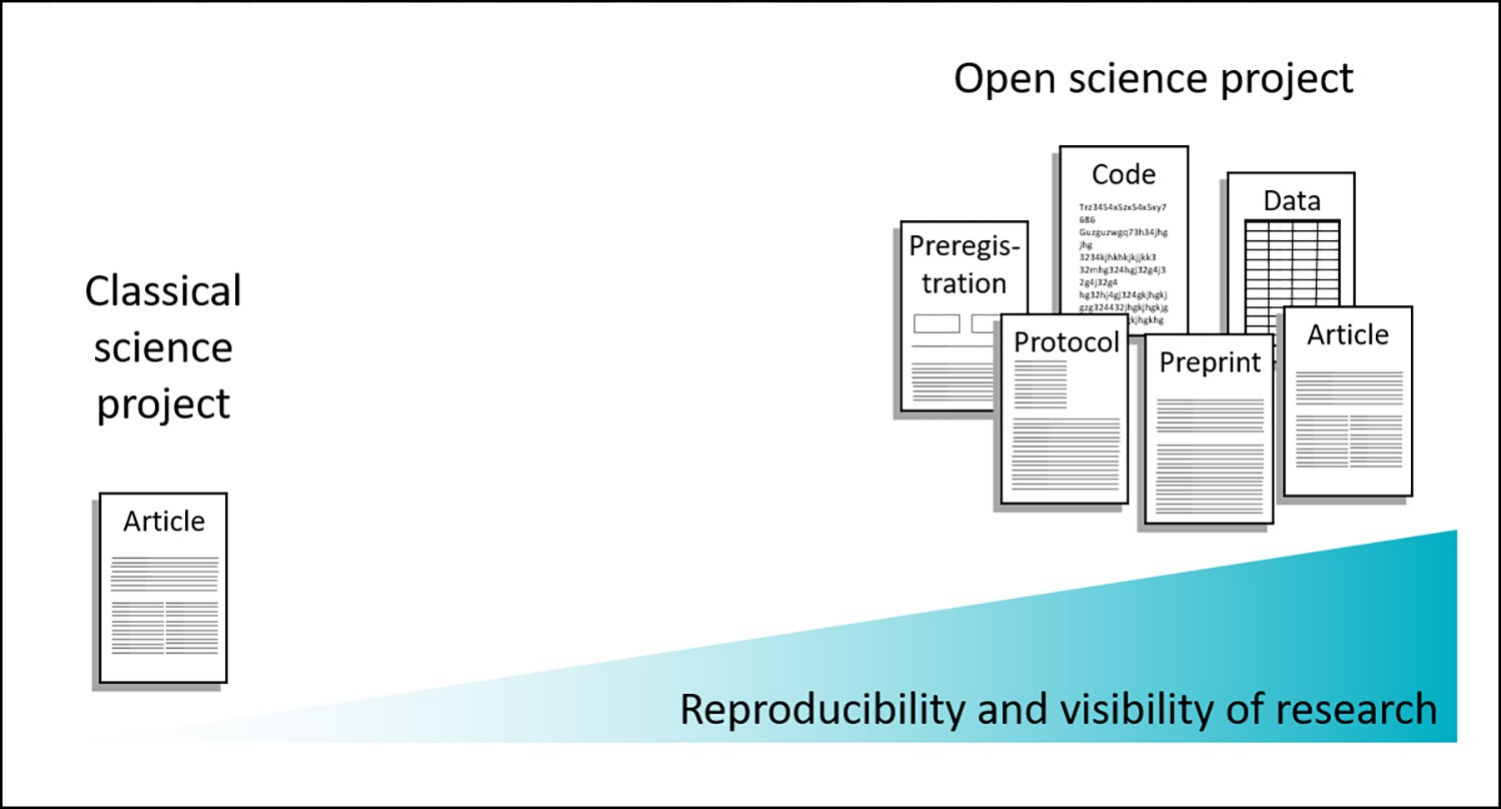
Published outcomes of classic versus open science projects. Application of open science practices can increase the reproducibility and visibility of a research project at the same time. By publishing different research outputs with more detailed information than can be included in a journal article, researchers enable peers to replicate their work. Reporting according to guidelines and using transparent visualization will further improve this reproducibility. The more research products that are generated, the more credit can be attributed. By communicating on social media or additionally publishing slides from delivered talks or posters, more attention can be raised. Additionally, publishing open access and making the work machine-findable makes it accessible to an even broader number of peers.

If scientists have used all available open science tools during the research process, then publishing and linking all outcomes represents the well-deserved harvest (Fig 2). At the end of a research process, researchers will not just have 1 publication in a journal. Instead, they might have a preregistration, a preprint, a publication in a journal, a dataset, and a protocol. Connecting these outcomes in a way that enables other scientists to better assess the results that link these publications will be key. There are many examples of good open science practices in laboratory animal science, but we want to highlight one of them to show how this could be achieved. Blenkuš and colleagues investigated how mild stress-induced hyperthermia can be assessed non-invasively by thermography in mice [103]. The study was preregistered with animalstudyregistry.org, which is referred to in their publication [104]. A deviation from the originally preregistered hypothesis was explained in the manuscript and the supplementary material was uploaded to figshare [105].

It might also be helpful to provide all resources from a project in a single repository such as Open Science Framework, which also implements other, different tools that might have been used, like GitHub or protocols.io.

### Communicating your research

Once all outcomes of the project are shared, it is time to address the targeted peers. Social media is an important instrument to connect research communities [106]. In particular, Twitter is an effective way to communicate research findings or related events to peers [107]. In addition, specialized platforms like ResearchGate can support the exchange of practical experiences (Table 1). When all resources related to a project are kept in one place, sharing this link is a straightforward way to reach out to fellow scientists.

With the increasing number of publications, science communication has become more important in recent years. Transparent science that communicates openly with the public contributes to strengthening society’s trust in research.

## Conclusions

Plenty of open science tools are already available and the number of tools is constantly growing. Translational biomedical researchers should seize this opportunity, as it could contribute to a significant improvement in the transparency of research and fulfil their ethical responsibility to maximize the impact of knowledge gained from animal experiments. Over and above this, open science practices also bear important direct benefits for the scientists themselves. Indeed, the implementation of these tools can increase the visibility of research and becomes increasingly important when applying for grants or in recruitment decisions. Already, more and more journals and funders require activities such as data sharing. Several institutions have established open science practices as evaluation criteria alongside publication lists, impact factor, and h-index for panels deciding on hiring or tenure [108]. For new adopters, it is not necessary to apply all available practices at once. Implementing single tools can be a safe approach to slowly improve the outreach and reproducibility of one’s own research. The more open science products that are generated, the more reproducible the work becomes, but also the more the visibility of a study increases (Fig 2).

As other research fields, such as social sciences, are already a step ahead in the implementation of open science practices, translational biomedicine can profit from their experiences [109]. We should thus keep in mind that open science comes with some risks that should be minimized early on. Indeed, the more open science practices become incentivized, the more researchers could be tempted to get a transparency quality label that might not be justified. When a study is based on a bad hypothesis or poor statistical planning, this cannot be fixed by preregistration, as prediction alone is not sufficient to validate an interpretation [110]. Furthermore, a boom of data sharing could disconnect data collectors and analysts, bearing the risk that researchers performing the analysis lack understanding of the data. The publication of datasets could also promote a “parasitic” use of a researcher’s data and lead to scooping of outcomes [111]. Stakeholders could counteract such a risk by promoting collaboration instead of competition.

During the COVID-19 pandemic, we have seen an explosion of preprint publications. This unseen acceleration of science might be the adequate response to a pandemic; however, the speeding up science in combination with the “publish or perish” culture could come at the expense of the quality of the publication. Nevertheless, a meta-analysis comparing the quality of reporting between preprints and peer-reviewed articles showed that the quality of reporting in preprints in the life sciences is at most slightly lower on average compared to peer-reviewed articles [112]. Additionally, preprints and social media have shown during this pandemic that a premature and overconfident communication of research results can be overinterpreted by journalists and raise unfounded hopes or fears in patients and relatives [113]. By being honest and open about the scope and limitations of the study and choosing communication channels carefully, researchers can avoid misinterpretation. It should be noted, however, that by releasing all methodological details and data in research fields such as viral engineering, where a dual use cannot be excluded, open science could increase biosecurity risk. Implementing access-controlled repositories, application programming interfaces, and a biosecurity risk assessment in the planning phase (i.e., by preregistration) could mitigate this threat [114].

Publishing in open access journals often involves higher publication costs, which makes it more difficult for institutes and universities from low-income countries to publish there [115]. Equity has been identified as a key aim of open science [116]. It is vital, therefore, that existing structural inequities in the scientific system are not unintentionally reinforced by open science practices. Early career researchers have been the main drivers of the open science movement in other fields even though they are often in vulnerable positions due to short contracts and hierarchical and strongly networked research environments. Supporting these early career researchers in adopting open science tools could significantly advance this change in research culture [117]. However, early career researchers can already benefit by publishing registered reports or preprints that can provide a publication much faster than conventional journal publications. Communication in social media can help them establish a network enabling new collaborations or follow-up positions.

Even though open science comes with some risks, the benefits easily overweigh these caveats. If a change towards more transparency is accompanied by the implementation of open science in the teaching curricula of the universities, most of the risks can be minimized [118]. Interestingly, we have observed that open science tools and infrastructure that are specific to animal research seem to mostly come from Europe. This may be because of strict regulations within Europe for animal experiments or because of a strong research focus in laboratory animal science along with targeted research funding in this region. Whatever the reason might be, it demonstrates the important role of research policy in accelerating the development towards 3Rs and open science.

Overall, it seems inevitable that open science will eventually prevail in translational biomedical research. Scientists should not wait for the slow-moving incentive framework to change their research habits, but should take pioneering roles in adopting open science tools and working towards more collaboration, transparency, and reproducibility.

## Abbreviations

CC: Creative Commons
CIRS-LAS: critical incident reporting system in laboratory animal science
COVID-19: Coronavirus Disease 2019
DOAJ: Directory of Open Access Journals
DOI: digital object identifier
EDA: Experimental Design Assistant
ELN: electronic laboratory notebook
EU: European Union
IMSR: International Mouse Strain Resource
JISC: Joint Information Systems Committee
LIMS: laboratory information management system
MGI: Mouse Genome Informatics
NC3Rs: National Centre for the Replacement, Refinement and Reduction of Animals in Research
NTS: non-technical summary
RRID: Research Resource Identifier
